# Mix it—cograzing with cattle reduces broiler losses and increases broiler range use

**DOI:** 10.1016/j.psj.2024.103906

**Published:** 2024-05-27

**Authors:** Severin Hübner, Lisa Schanz, Christoph Winckler, Kerstin Barth

**Affiliations:** ⁎ Institute of Organic Farming, Johann Heinrich von Thünen Institute, Federal Research Institute for Rural Areas, Westerau, Germany; † Institute of Livestock Sciences, Department of Sustainable Agricultural Systems, University of Natural Resources and Life Sciences, Vienna 1180, Austria

**Keywords:** broiler losses, mixed livestock, predatory bird, pasture use, broiler range use

## Abstract

Pasture access allows broilers to perform a wide range of behaviors and is a prerequisite in organic poultry production, but exposes broilers to various potential hazards including predators. Co-grazing broilers with cattle can reduce land use and could offer protection from avian predation. Thus, we aimed to assess the effects of co-grazing on broiler losses, range use, performance, contact dermatitis and broilers’ manipulation of cow pats. To this end, across 5 replicates we compared each a treatment group of 54 to 61 broilers co-grazing with 10 young cattle and a similar sized control group of broilers on a pasture which had been grazed by cattle 2 weeks prior. Broilers had pasture access during civil daylight and were locked in the coop overnight. Continuous video recordings of the pastures were used to identify the cause when broilers were missing or found dead. On 2 days per week in 4 replicates, broiler distribution in the pasture and maintenance behaviour (i.e. foraging, standing, lying, locomotion) were observed directly using instantaneous scan sampling. Based on the broilers’ distance to the coop we calculated a group Ranging Distance Index (**RDI**). Cow pats were assessed weekly and contact dermatitis was scored before slaughter. Broilers in the treatment groups ranged further (p = 0.003) and higher percentages of birds tended to be outside (p = 0.09) compared to the control groups. Broiler losses due to predatory birds were consistently lower in treatment (median, range: 1, 0 to 3) than in control groups (3, 2 to 5, p = 0.025). Live weight before slaughter was slightly higher (p = 0.035) in treatment groups than in control groups. Feed conversion ratio (p = 0.174), maintenance behaviors and prevalence of contact dermatitis were not affected. No manipulation of cow pats by broilers was found or observed. Overall, co-grazing with cattle positively affected broiler range use, losses due to avian predation and weight gain.

## Introduction

Access to an outdoor range provides chickens with additional space, enrichment and the opportunity to perform a wider range of natural behaviors compared to indoor housing systems (Dawkins et al., 2003). However, outdoor ranges may also impair chicken welfare e.g. by increasing the risk of disease (Hoop and Albicker-Rippinger, 1997; Permin et al., 1999) and predation (Bestman and Bikker-Ouwejan, 2020). Dutch organic laying hen farmers reported losses due to predatory birds or foxes of 3.7 %, with the majority being attributed to avian predation (Bestman and Bikker-Ouwejan, 2020). Such deaths occur along with mortality due to, for example, disease and cause monetary losses for farmers. These losses are usually not compensated by authorities as avian predation is not officially recognized as wildlife damage, i.e. predation by protected wildlife (Bestman and Bikker-Ouwejan, 2020).

Predation by foxes or other mammalian predators can be prevented by feasible measures such as locking chickens in a predator-safe house overnight and (electric) fencing of the range (Dawkins et al., 2003; Jones et al., 2007; Moberly et al., 2004). Countermeasures against avian predators, which usually hunt during the times when poultry has outdoor access, are often expensive, impractical and in some areas or countries subject to permits, e.g. netting of large outdoor ranges (Bestman and Bikker-Ouwejan, 2020). However, providing structures on the pasture such as bushes, trees and grass (and possibly artificial shelters) reduces poultry losses caused by avian predation (e.g. Dal Bosco et al., 2014). In addition, structures encourage range use and a more even distribution across the range (Dawkins et al., 2003). It has been shown that on outdoor ranges without structures, chickens tend to aggregate in or around the chicken house (Dawkins et al., 2003; Zeltner and Hirt, 2003). These aggregations can lead to damp and dirty bedding in the house and trampled and soiled forage around the house, which increases the risk of footpad dermatitis, hock burns and breast blisters (Ekstrand et al., 1998).

Within the EU, organic producers prefer slow-growing broilers as they are obliged to raise fast-growing broilers to at least 81 d of age (EU, 2018). Slow-growing broilers are more active (Bokkers and Koene, 2003), which may reduce aggregation, thereby reducing the risk of contact dermatitis. Even though slow-growing broilers have a higher locomotor activity level and are overall healthier (Rayner et al., 2020), range use may still be low (e.g. less than 15 % of the flock, Dawkins et al., 2003). The effect of different range conditions on body weight gain is unclear, as both higher (Ponte et al., 2008) and lower (Stadig et al., 2016; Sun et al., 2013) body weight has been found in slow-growing broilers with range access compared to indoor broilers.

Encouraging range visits, range use and foraging is essential for different aspects of chicken welfare, spanning from health to displaying more behaviors indicative of positive welfare (Rayner et al., 2020). Trees, shrubbery, and tall grass tended to be successful for increasing range use in chickens (i.e. number of chickens and duration outside as well as space use; Dawkins et al., 2003; Dal Bosco et al., 2014), possibly due to the reduced exposure to open space and lower risk of predation. Less successful in increasing range use were provision of forage, artificial shelters and enrichments (Riber et al., 2018).

Another strategy for increasing outdoor range use in chickens, which has received little attention to date, is to add another animal species to provide structure (Schanz et al., 2022), protection (Dal Bosco et al., 2014) and possibly an additional feed source through earthworms aggregated underneath cow pats (Bacher et al., 2018). Farms with more than one livestock species could benefit from implementing co-grazing strategies (Martin et al., 2020). In a survey of 102 organic mixed livestock farms from 7 European countries, cattle and poultry was one of the most prevalent species combinations with some farms implementing co-grazing (Ulukan et al., 2021). Presumed benefits, based on literature (Martin et al., 2020) and anecdotal evidence, are fewer poultry losses and increased range use, due to the presence of cattle, fostering animal health and weight gain.

To this end, the present study aimed to determine whether co-grazing broilers with cattle reduces broiler losses due to avian predation, increases broiler range use (i.e., number of broilers outside, distance to the coop), alters foraging habits (i.e., foraging in cattle dung pads) and improves health (i.e., footpad dermatitis, breast blisters and hock burns) and productivity (i.e., broiler weight, feed conversion)

Our hypotheses were: (1) Broiler groups on pasture with cattle experience fewer losses due to predatory birds compared to control groups with only broilers. (2) More broilers are outside their coop and at a greater distance to the coop in groups with cattle compared to groups with only broilers. (3) Broilers explore cattle dung pads when foraging in both treatment and control groups. (4) Fewer broilers exhibit contact dermatitis when on pasture with cattle compared to broilers on pasture with only conspecifics. (5) Broilers on pasture with cattle have a higher weight at slaughter and better feed conversion than broilers on pasture with only conspecifics.

## MATERIAL AND METHODS

The experiment took place between 2019 and 2021 at the Thünen Institute of Organic Farming in Westerau, Germany. Every year we conducted 2 trials between June and October, except for 2021 when only one trial took place between June and August. The Ministry of Energy, Agriculture, the Environment, Nature and Digitalization in Schleswig-Holstein approved all procedures related to this study (reference V242-46376/2019; V242-26697/2021).

### Experimental Setup

In 5 six-week replicates with in total 10 groups of broilers (each 54–61 birds), we allocated one group to treatment and one to control per replicate. Broilers assigned to the treatment groups spent six weeks on pasture co-grazing with 10 young dairy cattle, whereas the control groups spent 6 wk on pasture which had been grazed by a second cattle group 2 wk prior to the broilers’ arrival. The second cattle group was included to also improve the comparability between both systems.

No (artificial) shelters were provided to broilers to determine the effect of the presence of cattle on broiler behavior. In the first trial, broilers were randomly assigned to treatment or control groups, whereas broilers in the last 4 trials were assigned to groups based on their results in a Tonic Immobility test creating 2 groups with the same average duration spent in tonic immobility (Schanz et al., 2022). Additional data from pilot studies with a slightly different setup (e.g., smaller pop holes, artificial shelters in the control groups) can be found in the supplementary material (Supplementary Material S1).

**Figure 1:**
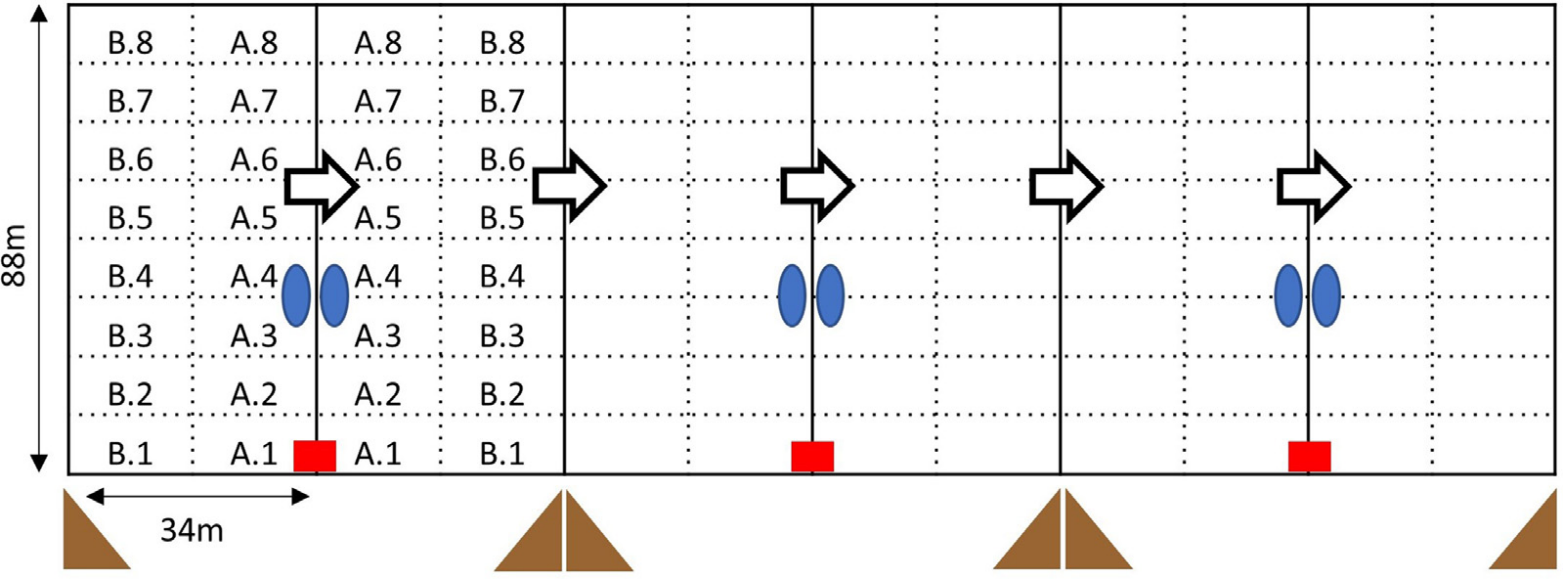
Experimental set up in a rotational pasture system with 6 plots. Dashed lines indicate sectors used for localization of animals during observations, each measuring 11 m x 17 m. The red rectangle represents the broilers’ coop with access to the adjacent plots. Blue circles represent the drinking trough for cattle which were also set up in the control group and brown triangles the observer's and camera's location. The arrows indicate the weekly rotation to the next plot.

Groups were assigned to pastures in a balanced order across trials and seasons. The two pastures were 300 m apart and separated by several buildings and high vegetation. Each pasture (1.8 ha) was subdivided into 6 plots of equal size (0.3 ha) and groups were moved to a new plot every week (Figure 1). A portable poultry netting (not electrified) with an edge length of 9 cm per mesh was used. For the control groups the poultry netting was placed inside the cattle fence, whereas for the treatment groups it was placed outside the cattle fence to avoid cattle getting tangled in the poultry netting.

The chicken coop could be opened on 2 sides and was located at the intersection of two plots (Figure 1), thereby the active pop hole (width: 80 cm) could be switched to allow access to either plot and the coop was only moved every second week. The pop-hole was controlled by light sensors set to open and close at the beginning and end of civil daylight respectively.

### Animals

In total 575 slow-growing hybrid broilers (54–61 broilers x 2 groups x 5 trials) and 60 young cattle (20 cattle per year x 3 years) were part of this experiment. The broiler strain (Hubbard JA 757) was officially recognized as suitable for organic farming in Germany as the expected daily weight gain is below 80 % of weight gain in common commercial hybrid broilers (Hörning et al., 2010). Broilers were purchased from a certified organic hatchery (Geflügelhof Overmeyer, Hopsten-Halverde), vaccinated against Marek's disease and coccidiosis (Novilis Rismavac, Paracox 8, MSD Animal Health) as one-day-old chicks.

Before given access to pasture, the broiler chicks were raised for 30 d in mixed-sex groups (108–125 broilers) in an indoor area (3.4 × 6.8 m) with a stocking density of 4.7 to 5.4 animals/m² and an adjacent covered outdoor area (3.1 × 11.3 m), to which broilers had access from the age of 14 d onwards (stocking density: 3.1–3.6 animals/m^2^). Mortality rates in the first 30 d were between 0 and 3 chicks and occurred within the first 3 d after arrival.

From d 1 chicks had ad libitum access to pelleted feed (Unimastfutter Eiderkraft, Gut Rosenkrantz Bio-Futter GmbH & Co KG, CP 19.5%, ME/kg 11.4 MJ) of which 10, 15, 20, and 30% were substituted with wheat starting at the age of 14, 21, 28 and 37 d respectively. At the age of 20 d, chicks were gradually accustomed to the treadle feeders (Siepmann, Germany) used throughout the experiment.

At the age of 30 d, broilers were assigned to treatment and control groups and moved to pasture. On pasture, broilers had access to a straw-bedded coop with perches (Atlantic Systeme GmbH, 3.0 × 4.6 m) and ad libitum access to feed and water (2 hanging low pressure drinkers with 10 nipples each, Siepmann). At the age of 73 d, broilers were slaughtered at a certified organic slaughterhouse. Broilers were electrically stunned with a current of 400 mA to the head and then killed by decapitation.

Cattle had an average age of 6.1 ± 1.4 mo (mean ± standard error of the mean) at the beginning of the June to July – trials each year and 8 ± 1.2 mo at the beginning of the September to October – trials. They were assigned to treatment and control groups based on their weight, creating 2 groups with a similar average weight (June to July – trial: 191 ± 8.1 kg, Sept. – Oct. trial: 226 ± 8.3 kg). Young cattle were derived from the research farm's dairy herd and had been raised indoors. Cattle were gradually habituated to pasture starting 2 wk prior to the start of the first trial each year. During the experiment cattle were continuously on pasture with ad libitum access to fresh water (200 l trough WT 200, Suevia Haiges GmbH, Germany) as well as a mineral block (JOSERA Rindereimer, Josera GmbH & Co. KG, Germany) and were fed concentrate once a day in troughs (0.5 kg per animal).

### Broiler Losses

Carcasses found on pasture were documented on a daily basis and missing birds at the time of each weighing. The cause of death and reason for missing birds was determined using video recordings. One camera per pasture (Panasonic HX-WA30, Anpviz PoE IP Camera Outdoor) continuously recorded the whole range area to which broilers had access including the pop holes of the coop.

### Range Use and Maintenance Behaviors

Range use and maintenance behaviors (i.e., foraging or pecking at ground, lying, standing and in locomotion) of broilers were directly assessed via instantaneous scan sampling. Each plot was visually split once on the short side and 7 times on the long side, resulting in 16 equally sized sectors measuring 11 × 17 m (Figure 1). The number of broilers in each sector was counted every 6 minutes (21 scans per 120 min observation) in 2 observation periods per day. Broiler behavior was recorded in terms of 4 maintenance behaviors at the time of each scan (see Table 1 for ethogram). Observations took place during the early morning (shortly after the beginning of civil daylight) and late evening (before the end of civil daylight), as previous research suggests high activity in chickens during dusk and dawn (Savory, 1976). In the first trial two observers rated the same group at the same time to assess inter-observer agreement for number of broilers outside, broilers in each sector and behaviors. To assess the effect of co-grazing with cattle, simultaneous observations across the last 4 replicates were used to exclude possible influencing factors specific to individual observation periods. Of the 96 planned observation periods (2 observations per day x 2 d per week x 6 wk x 4 trials), n = 87 observations per group were included in the analysis (9 observations could not be used due to unforeseen events during these periods, e.g. severe weather conditions).

**Table 1:** Ethogram for broiler maintenance behaviors recorded using scan sampling (Schanz et al., 2022).

Behavior	Description
Lying	Broiler sits with both legs bent and abdomen in contact with ground or on side (i.e., including any behavior conducted lying).
Standing	Broiler is not lying, supported by its legs and not in motion; no other body part is touching the ground including any behavior conducted standing.
Foraging	Broiler is consuming or manipulating vegetal substrate (pecking at ground).
Locomotion	Broiler is walking or running (including running steps with both feet in the air).

To assess a possible effect of weather on broilers’ maintenance behavior and range use, solar radiation, wind speed, precipitation, air temperature and humidity were recorded in 30 min intervals at a weather station approximately 1 km from the study site. Precipitation was not included in the analysis due to frequent mismatches between the weather station's recordings and personal notes during observation.

### Assessment of Cow Pats

To investigate whether broilers use cow pats as a feed source, the pastures were walked by one assessor the day before animals were moved to the next plot in both treatment and control groups (grazed by cattle 2 wk prior to broilers arrival). Dung pats placed on the pastures were of different ages, that is, 0 to 7-days-old for the treatment groups and 14 to 21-days-old for the control groups. The cow pats were scanned for scratch marks and photographed. In total 480 cow pats were investigated.

### Live Weight, Feed Conversion, and Contact Dermatitis

The amount of feed added to feeders and the remaining feed before refilling was recorded once a week and at the end of the trial, allowing the calculation of feed consumption per group and week. Broilers were weighed individually every second week, when the coop was moved, and on the day before slaughter. At slaughter we assessed each broiler for the occurrence of footpad dermatitis, hock burns and breast blisters using an adapted version of the Welfare Quality Protocol (Butterworth et al., 2009; Jong et al., 2016) (Table 2).

**Table 2:** Scoring system for the assessment of animal welfare indicators at slaughter: footpad dermatitis, hock burns and breast blisters with their levels and descriptions (Jong et al., 2016).

Indicator	Scores	Description
Footpad dermatitis	0	No or only a very small area (1 – 2 mm) affected, light coloration or healed scars
	1	Clear discoloration (> 2 mm), small ulcerations (1 – 2 mm)
	2	Deep ulceration, strongly swollen footpad
Hock burns	0	No dermatitis, at most light coloration
	1	Clear discoloration, swollen hocks
	2	Necrotic lesions, black discoloration
Breast blisters	0	No breast blister on carcass
	1	Breast blister on carcass containing liquid

### Inter-Observer Agreement

We assessed the inter-observer agreement between two observers for the percentage of broilers outside, the number of broilers per sector and the percentage of broilers performing the 4 maintenance behaviors (n = 19 observations, 2 h duration). As a measure for agreement, we calculated the Intraclass Correlation Coefficient (**ICC**) with the 95 % confidence intervals (**CI**) based on single measurements, absolute agreement and a 2-way model. ICC values above 0.9 indicate excellent, values between 0.9 and 0.75 good and values between 0.75 and 0.5 moderate agreement (Koo and Li, 2016).

For the total numbers of broilers outside the ICC was excellent (lower CI < ICC < upper CI, 0.925 < 0.97 < 0.99, F(15, 15.4) = 69.7, *p* < 0.0001). During observations for the assessment of inter-observer agreement regarding the number of broilers per sector, broilers were only observed in sectors A.1, A.2, B.1, B.2 and B.3 sufficiently often to calculate the ICC, resulting in good to excellent agreement (Supplementary Material S2). Agreement for broiler maintenance behaviors was excellent for lying, good for foraging and standing and moderate for locomotion (Supplementary Material S3).

### Data Analysis

Broiler losses due to predatory birds and other causes in control and treatment groups are reported as medians for each replicate. Due to the non-normal distribution of the data, Friedman's test was conducted on the 5 trials to examine the effect of the presence of cattle on broiler losses. Additionally, we compared the ratio of mean rates between groups (risk ratio), which is based on the odds ratio of relative losses in the treatment and control groups.

To assess range use, the total number of broilers outside and the broilers’ distance to the chicken coop based on the number of broilers in each sector were computed. The percentage of broilers outside was calculated per observation based on the percentages of birds outside the coop in the 21 scans per observation. We used simultaneous observations from 4 trials to describe range use across time and to assess the difference in range use between treatment and control groups. The difference was calculated by subtracting the percentage of broilers outside in the control group from the percentages of broilers outside in the respective treatment group. To test for statistical significance, we used a paired t-test. The sample size was too low for further statistical analysis and we thus used descriptive statistics as well as data visualizations to describe range use. The ggplot2 package (Wickham, 2016) was used for all visualizations. To assess range use we calculated a collective ranging distance index (**RDI**) based on each sector's distance to the coop and the respective number of broilers in it (adapted from the individual ranging distance index by Ferreira et al., 2020). We assume that each broiler recorded in one sector has on average traversed some distance in this sector and we are using the geometric middle of the sector as the reference. Due to our set-up (coop in one corner of the plot) and the rectangular shape of our sectors (Figure 1), we calculated one value for each sector based on the direct distance between the geometric middle point of this sector and the coop. For example, for a broiler recorded in sector B.3 we calculated the distance with the Pythagoras theorem based on the length of two and a half sectors (2.5 × 11 m) and the width of one and a half sectors (1.5 × 17 m) resulting in 37.5 m. We postulate that each broiler has traversed at least the direct distance between the coop and the sector it is recorded in. For the RDI we multiplied the number of broilers with the calculated distance per sector and summarized per scan:

$$\sum_{i=1,\;j=a=0}^{i=8,\;j=b=1}nb\;of\;broilers\;in\;sector\;ij\;\times \;\sqrt{{\left(\left(i-1\right)\times L+\frac{L}{2}\right)}^{2}+\;{\left(j\;\times W+\;\frac{W}{2}\right)}^{2}},$$

where i was the sector number and j the sector letter (a = 0 and b = 1). L corresponds to the sector length (11 m) and W to its width (17 m). The occurrence of broilers foraging, lying, standing and in motion were expressed as the mean percentage of birds outside performing the respective behavior (i.e., the number of broilers recorded performing one behavior divided by the total number of broilers outside) and a paired t-test was used to test statistically significant differences between groups.

To assess the possible association of weather conditions such as solar radiation, wind speed, air temperature and humidity with the percentage of broilers outside the coop or percentage of broilers performing one behavior (foraging, lying, standing and locomotion) a Pearson's correlation coefficient was used. For this analysis, the mean of 5 consecutive recording intervals (each 30 min) from the weather station were matched with the corresponding recordings from scan sampling for each maintenance behavior (i.e., foraging, lying, standing, locomotion) and range use variable (i.e., percentage broilers outside and RDI) Weather recordings for 80 observation periods were available (7 could not be used due to a malfunction of the weather station).

Feed Conversion Ratio (**FCR**, kg feed/kg live weight before slaughter) was calculated per group from the start of the pasture period (age 30 d) until the day before slaughter (age 72 d). For deceased broilers an estimated feed consumption for the days they had been alive after the last weighing of feed was included in the calculation to account for all broilers consuming feed. This estimation was based on the number of days alive after the last weighing multiplied by the average daily feed consumed in the respective group (treatment or control), trial and week.

Since food pad dermatitis and hock burns were rarely scored at the highest of three levels (score 2) and breast blister only had 2 levels, we created binary variables for all 3 indicators (i.e., whether a contact dermatitis was present or not). Scores were expressed as percentage of broilers with footpad dermatitis, hock burns and breast blisters per group.

For broiler live weight before slaughter (age 72 d, n = 525), FCR and the percentage of affected broilers per contact dermatitis severity level, we calculated the mean per group and replicate (n = 5) and performed paired t-tests. All statistical analyses were done in the statistical programming language R (version 4.2.2, (R Core Team, 2022), RStudio version 2023.3.0, RStudio Team, 2023). The assumption of normal distribution of residuals was verified by visually inspecting QQ plots.

## RESULTS

### Broiler Losses

Of the 575 broilers included in the trials, 50 died before slaughter. Twenty-six deaths could be attributed to predatory birds and 21 to foxes. In the control groups (only broilers), losses due to avian predators were higher than in the groups of broilers ranging with cattle (*χ²(1) = 5, p = 0.025*), amounting to a median across trials of 4 broilers in control groups and 1 broiler in treatment groups (for details see Table 3). It was 3.4 times more likely (risk ratio) for a broiler in the control groups to be killed by an avian predator than in the treatment groups.

**Table 3:** Number (and percentage) of broiler losses due to avian predation and other causes for control and treatment groups in each trial. Percentages of broiler losses are calculated based on the flock size at the start of each trial.

Year	nb	Months	Predatory birds		Other causes		Total nb broilers	
			Treatment	Control	Treatment	Control	Treatment	Control
2019	1	June-July	0	2 (3.6)	0	1 (1.8)	55	55
	2	Sept-Oct	1 (1.8)	5 (8.6)	2 (3.6)	1 (1.7)	55	58
2020	3	June-July	1 (1.9)	2 (3.7)	0	5 (9.3)^1^	54	54
	4	Sept-Oct	3 (4.9)	4 (6.6)	16 (26.2)^1^	0	61	61
2021	5	June-July	0	3 (4.9)	0	1 (1.6)	61	61
Total			5	16	18	8	286	289

1 Losses due to foxes.

For 19 out of 26 broiler losses due to avian predators, video recordings were available. In 18 cases European Goshawks (*Accipiter gentilis*) and in one a common buzzard (*Buteo buteo*) were identified. For one case, the type of avian predator could not be identified. For the remaining 7 deaths no video recordings were available, but the carcasses showed clear signs of an attack by a predatory bird including torn feathers, isolated holes of claws and the absence of paired-bite marks (Stahl et al., 2002). Additionally, in these 7 instances only one broiler was killed at a time, which is common for avian predators, but not mammalian predators.

In 2020 two fox attacks occurred, killing 21 birds in total. Fox predation occurred in the early morning (around 2 am and 5 am) in presence and absence of cattle (for details on the number of deaths see Table 3). Video recordings showed that broilers had not entered the coop before the automatic door closed at the end of civil daylight the previous evening and were thereby exposed to the fox attacks.

The remaining losses could either not be attributed to a specific predator due to ambiguous marks on the carcass (n = 1) or no external impacts were visible (sudden death, n = 1) or broilers left the fenced area without returning (n = 1).

### Range Use

**Figure 2:**
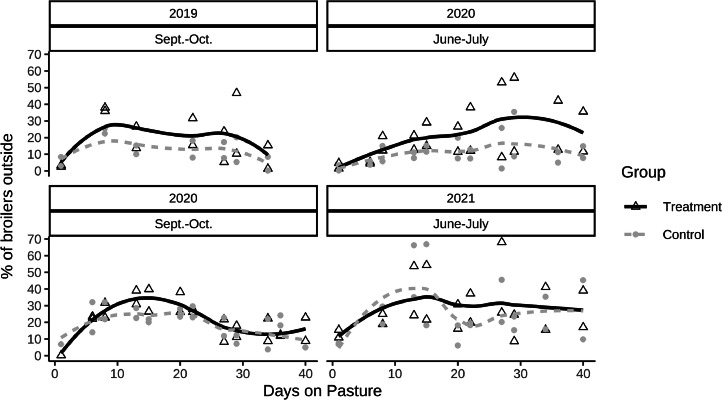
Percentage of broilers outside in treatment and control groups across time (6 wk) for 4 replicates with 4 simultaneous observations per day (n = 158).

The average percentage of broilers outside the coop (during the 2h-observation periods) was (mean ± standard error of mean): 22.7 ± 1.6 % in the treatment groups and 16.8 ± 3.4 % in the control groups (*t(3) = 2.5, P = 0.09*). Range use changed over time and differed between morning and evening observations. The percentage of broilers outside during observations on the first 3 days on pasture was below 15 %, increased in the first 2 wk on pasture in both treatment and control groups, almost plateaued and decreased slowly in the last 2 wk on pasture (Figure 2). The percentage of broilers outside the coop within an hour in both groups ranged from 0 to 75%. The largest percentage of broilers seen outside during observations was 95% of a treatment group. The highest percentages of broilers outside the coop were usually recorded in the evening and in the treatment groups. The lowest percentages of broilers outside the coop were recorded in the morning with a minimum of 1 broiler being recorded outside in 120 minutes (21 scans) usually in the control groups.

**Figure 3:**
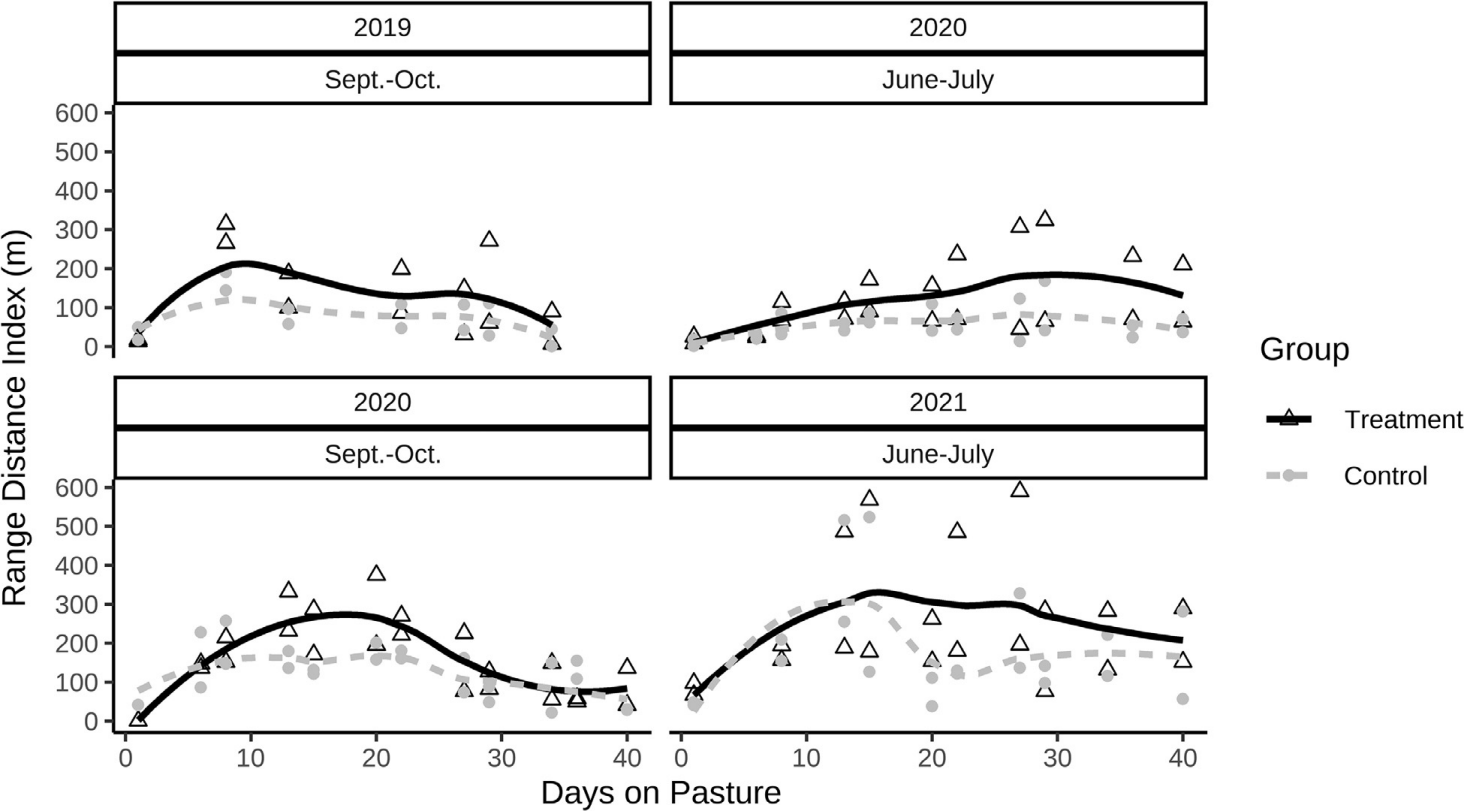
Ranging Distance Index in treatment and control groups across time (6 wk) for 4 replicates with 4 simultaneous observations per day (n = 158).

Similar to the percentage of broilers outside, broilers’ distance to the coop increased in the first 2 wk and decreased over time. The mean Ranging Distance Index was 164 ± 26 m (range of means per observation period: 1–570 m) in the treatment groups and 111 ± 28 m (range: 0–523 m) in the control groups (*t(3) = 8.8, P = 0.003,* Figure 3). The highest value in a single scan was observed in the treatment groups with 1204 m. Most of the broilers outside were observed close to the coop (within 11 m): 87.2 ± 3.4% in the treatment groups and 93.1 ± 1.8% in the control groups. In the treatment groups there were 7 occurrences of broilers being observed at a greater distance to the coop than 66 meters (sectors 7 or 8; at least 1 occurrence per replicate except in the June to July trial 2020).

**Figure 4:**
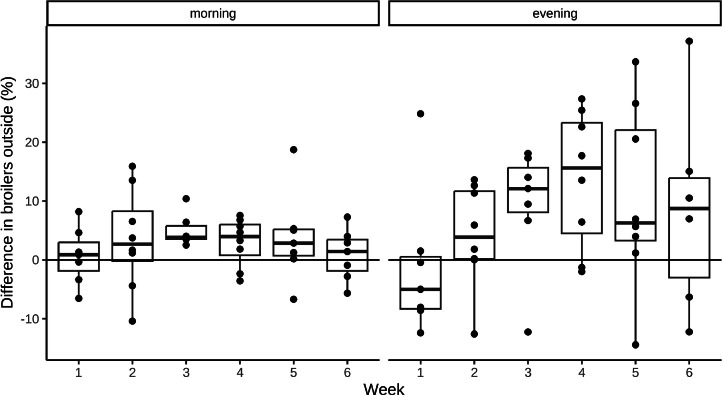
Differences in percentage of broilers outside during morning and evening observations (based on 174 simultaneous observations, n_max_= 8, 4 trials x 2 observations per morning and evening). Positive values indicate more broilers outside in the treatment group, whereas negative values indicate a higher percentage of broilers outside in the control group.

Differences between groups were less pronounced during morning observations (15 % vs. 8.2 %, *t(3)* = *2.15, P = 0.121*) than evening observations (30.4 % vs. 22.0 %, *t(3) = 3.75, P = 0.033,* Figure 4). The relative difference between groups for the RDI was similar in both morning (107 ± 12 m vs 78 ± 9 m, *t(3) = 6.13, P = 0.009*) and evening observations (221 ± 20 m vs. 144 ± 17 m, *t(3) = 4.83, P = 0.017*).

### Maintenance Behavior

On average, in both treatment and control groups 73 to 75% of broilers outside were recorded foraging (Table 4). The control groups on average had more broilers standing or moving than the treatment groups. There were no to only moderate correlations between different weather indicators (i.e., solar radiation, wind speed, air temperature and humidity) and range use or maintenance behaviors (Supplementary Material S4).

**Table 4:** Percentage of broilers (mean ± sem) outside performing maintenance behaviors pooled per replicate (n = 4).

	Control	Treatment	Statistical test	p-value
Lying	12.6 ± 4.0	18.1 ± 5.1	*t (3) = 1.30*	*0.284*
Standing	8.2 ± 0.8	3.9 ± 1.0	*t (3) = 4.49*	*0.021*
Foraging	73.1 ± 4.3	74.7 ± 5.3	*t (3) = 0.34*	*0.757*
Locomotion	6.2 ± 0.6	3.3 ± 0.2	*t (3) = 5.16*	*0.014*

### Cow Pats

None of the 480 investigated cow pats showed any scratch marks in neither treatment nor control groups. Anecdotally, only in the last trial 1 broiler was seen pecking beetles from a cow pat. Broilers were not observed foraging in cow pats but predation of insects was observed during foraging events.

### Live Weight and Feed Conversion

Live weight at the beginning of the trials did not differ between groups (0.84 ± 0.04 kg, Supplementary Material S5). Before slaughter, it was higher in treatment groups (3.1 ± 0.07 kg) than in control groups (LW: 3.0 ± 0.09 kg, *t(4) = 3.13, P = 0.035*). The feed conversion ratio (from start of the pasture period until the day before slaughter) did not differ between treatment (3.0 ± 0.17) and control groups (3.3 ± 0.21; *t(4) = 1.65, P = 0.174*).

### Contact Dermatitis

The prevalence of footpad dermatitis, hock burns and breast blisters was highly variable between the trials and did not consistently differ between treatment and control groups (see Table 5).

**Table 5:** Prevalence (mean ± sem) of the animal welfare indicators footpad dermatitis, hock burns and breast blisters (% of flock affected) at slaughter, based on the average values of each replicate (n = 5).

	Control	Treatment	Statistical test	p-value
Footpad dermatitis	15.2 ± 7.1	8.9 ± 3.8	*t (4) = 1.48*	*p = 0.214*
Hock burns	2.8 ± 2.1	1.8 ± 0.6	*t (4) = 0.51*	*p = 0.632*
Breast blisters	5.9 ± 1.7	6.2 ± 1.8	*t (4) = 0.13*	*p = 0.901*

## DISCUSSION

The aim of our study was to investigate the effects of co-grazing cattle and broilers on broiler losses due to avian predation, range use, foraging habits as well as aspects of health and productivity. The nature of such a system (including poultry rearing into a rotational grazing system) allowed us to conduct only 5 replicates of which one had to be used for determining the inter-observer-agreement. The number of broilers in each sector of the range, the percentage of broilers outside and their behavior could be reliably assessed by 2 independent observers. The presence of cattle coincided with fewer broiler losses due to avian predators and a greater percentage of broilers outside and further away from the coop. More broilers were lying down in the treatment groups than in the control groups; none of the broilers were observed pecking at cow pats. Broilers grazing with cattle had a slightly higher final weight compared to control groups with only broilers on pasture. The incidence of contact dermatitis was similar in both groups.

We interpret the consistently fewer broiler losses due to avian predators in groups co-grazing with cattle, in which more broilers were outside, as a result of the cattle's presence. This could be due to cattle unpredictably moving in the area with broilers or due to cattle being perceived as obstacles obscuring the line of sight or flight path for attack by predatory birds. It is unclear whether avian predators refrained from attacking in the presence of cattle or if attacks were interrupted by the presence of cattle. The number of unsuccessful attacks could yield some insights but is unknown in our study. Future studies investigating this aspect of avian predation could help determine the size and type of animals or objects suitable to reduce losses to avian predators. Anecdotal evidence suggests that stationary objects with moving components (e.g., strips of fabric) deter avian predators only for short periods of time, as these predators quickly habituate to the new stimuli and their predictability (Keil, 1962; Deerberg, 2014; Freeman, 2022). It is also possible that the presence of cattle influenced the behavior of broilers and possibly their reaction to an attack by a predatory bird. However, as the majority of broiler behaviors have been shown to not be affected by the presence of cattle (Schanz et al., 2022), we deem this less likely. With approximately 5.5% losses due to avian predation in the control groups, losses in our study were lower compared to an earlier study reporting 9.7% losses due to avian predation (Otto, 1980). They were, however, in a similar range as the losses due to total predation with the majority attributed to predatory birds reported in other studies (Stahl et al., 2002; Bazer, 2005; Ponte et al. 2008; Bestman and Bikker-Ouwejan, 2020). In accordance with the only other study specifying the species of predatory birds responsible for poultry losses (Bestman and Bikker-Ouwejan, 2020) most avian attacks in our study were conducted by goshawks except for 2 conducted by buzzards. The total mortality rates in treatment (8.0%) and control groups (8.3%) were in a similar range as other experimental and survey studies on free-range farms (3–8.4%; Sommer, 2001; Stahl et al., 2002; Schmidt et al., 2009; Hörning et al., 2010).

In line with our hypotheses, broilers co-grazing with cattle were outside the coop in greater numbers (22.7 %) and ranged further (RDI 164 m) than their conspecifics in the control groups (16.8%, RDI 111 m). The Ranging Distance Index is based on the direct distance between a sector's middle point and the coop, but broilers usually took a more circuitous route to the sector they were recorded in. Therefore, the index is an indication of the groups’ average distance to the coop. As the index does not differentiate for example, between one broiler in sector 8 and eight broilers in sector 1, its explanatory power is limited. Nevertheless, the RDI describes range use of a group better than just reporting the maximum distance of individual broilers. In addition, observer perception is more accurately represented by the RDI. Given that avian predation losses were lower in treatment groups, broilers may have perceived the outdoor range as safer due to few (successful) attacks compared to broilers in control groups. Additionally, broilers possibly perceive cattle as a structural element on the pasture and therefore venture further (Schanz et al., 2022). One study using woody plantation found a similar percentage of broilers outside in treatment (22.4%) and control (16.3%) groups (Jones et al., 2007) as in our study. Observation times and protocols were different to ours possibly masking different effects. Artificial shelters and shading structures did however not increase the percentage of broilers outside (Rodriguez-Aurrekoetxea et al., 2014). Our results may indicate that co-grazing with cattle has a similar effect on the percentage of broilers outside as woody plantation and possibly a greater effect than artificial shelters. The higher percentage of broilers standing or in locomotion in the control groups could be interpreted as an indicator of higher vigilance due to more frequent attacks by predatory birds or the lack of structures offering protection, but further research is necessary to corroborate this tentative interpretation.

Broilers in our study were not observed pecking at dung pads and dung pads did not show signs of pecking or scratching by broilers, which is in line with the only other study investigating this behavior in broilers (Phillips et al., 2020). Laying hens have been observed pecking at and spreading cattle dung over larger area, facilitating a more even fertilization of the soil (Nikol, Gut Rothenhausen, Schleswig Holstein, personal communication). Ferreira et al. (2021) have previously shown that individuals ranging more frequently and further also spent more time searching for feed. Those high ranging individuals even preferred feed they had to search for over readily available feed – a concept also known as “contrafreeloading.” This could also extend to feed sources in and around cow pats. Higher ranging animals could be achieved by changing the feeding regimen (Horsted et al., 2007) and use of different breeds for example, Bresse (Almeida et al., 2012; Bonnefous et al., 2023) or Barred Plymouth Rock (Clark and Gage, 1996).

There was no difference in the incidence of footpad dermatitis, hock burns and breast blisters between treatment and control groups. We expected fewer contact dermatitis in broilers co-grazing with cattle compared to the control groups, due to the expected greater range use and mobility, decreasing the time broilers spent lying down in damp or dirty areas (Castellini et al., 2016). There were however no notable damp areas during the trials. Broilers co-grazing with cattle spent less time lying and more time standing or in motion, but this did not affect skin lesion prevalence, possibly due to the difference being small or the possibly greater impact of breed (Castellini et al., 2016). The incidence of footpad dermatitis which was generally of a low severity level was not positively affected by more broilers being outside or ranging further when on pasture with cattle (9.8%) compared to broilers in the control groups (11.9%).

Weight before slaughter was slightly higher (3.1 kg) in groups co-grazing with cattle compared to control groups with only broilers (weight 3.0 kg). Weight gain could be positively influenced by broilers foraging additional feed when spending more time outside their coop in treatment groups and thereby influencing the development of the gastro-intestinal tract, possibly increasing nutrient absorption (Marchewka et al., 2021). However, other studies did not find differences in weight between groups with different ranging behavior (Stadig et al., 2016; Dal Bosco et al., 2014), which could indicate that the weight difference in our study was a result of factors other than time spent outside. For the FCR no differences could be found between treatment and control groups which is in line with findings by Ponte et al. (2008) who found higher LW but unchanged FCR in free range slow-growing broilers.

## CONCLUSIONS

We conclude that co-grazing with cattle can be beneficial for broilers in terms of increased range use, fewer losses due to avian predators and possibly higher weight gain, and thereby presents a possibly desirable management strategy for farmers with cattle and poultry. Further research is necessary to determine the (external) validity of these findings based on a greater sample size (number of groups) as well as the applicability with a higher number of broilers per group reflecting commercial conditions.

## DISCLOSURES

The authors declare no conflicts of interest.

## Appendix Supplementary materials

Image, application 1

## Footnotes

Supplementary material associated with this article can be found in the online version at doi:10.1016/j.psj.2024.103906.
