# Supplementary material for: Mix it—cograzing with cattle reduces broiler losses and increases broiler range use
Source: Poult Sci. 2024 May 27;103(8):103906. doi: 10.1016/j.psj.2024.103906 (PMC11234040; doi:10.1016/j.psj.2024.103906)
Supplement: Supplementary file 1 [file mmc1.docx]

**Mix it – Co-grazing with cattle reduces broiler losses and increases broiler range use**

Severin Hübner, Lisa Schanz, Kerstin Barth, Christoph Winkler

**Supplementary Material**

**S1.1 Table** Number (and percentage) of broiler losses due to avian predation and other causes for control and treatment groups in a pilot study of 2018.

| Year | nb | Months | Predatory birds | | Other causes | | Total nb broilers | |
| --- | --- | --- | --- | --- | --- | --- | --- | --- |
|  |  |  | Treatment | Control | Treatment | Control | Treatment | Control |
| 2018 | 1 | June-July | 0 | 3 (5.5) | 0 | 0 | 55 | 55 |
|  | 2 | Sept-Oct | 2 (3.6) | 5 (8.9) | 1 (1.8) | 1 (1.8) | 55 | 56 |
| Total |  |  | 2 | 8 | 1 | 1 | 110 | 111 |

**S1.2 Table** Prevalence (mean ± sem) of the animal welfare indicators footpad dermatitis, hock burns and breast blisters (% of flock affected) at slaughter, based on the average values of two replicates in a pilot study of 2018.

|  | Control | Treatment |
| --- | --- | --- |
| Footpad dermatitis | 3.65 ± 2.45 | 12 ± 4.3 |
| Hock burns | 5.95 ± 1.85 | 7.45 ± 1.65 |
| Breast blisters | 17.2 ± 9.35 | 13.6 ± 11.9 |

In the pilot study, 10 artificial shelters were placed within a 25-meter radius from the coop in the control group. The shelters were removed for the experiment 2019-2021 to extrapolate the influence of cattle on pasture. In the first trial the width of the pop-hole was 40 cm. As this led to individual broilers blocking the entrance, from the second trial of the pilot study (and also the subsequent experiment) the width was adjusted from 40 cm to 80 cm. Due to a draught in the second trial of the pilot study the cattle groups were reduced from 10 to 5 animals per group.

**S2 Figure:** Inter-observer agreement between the 2 observers for the number of broilers per sector assessed with the Intraclass Correlation Coefficient (ICC, dot) and confidence intervals (CI, whiskers).
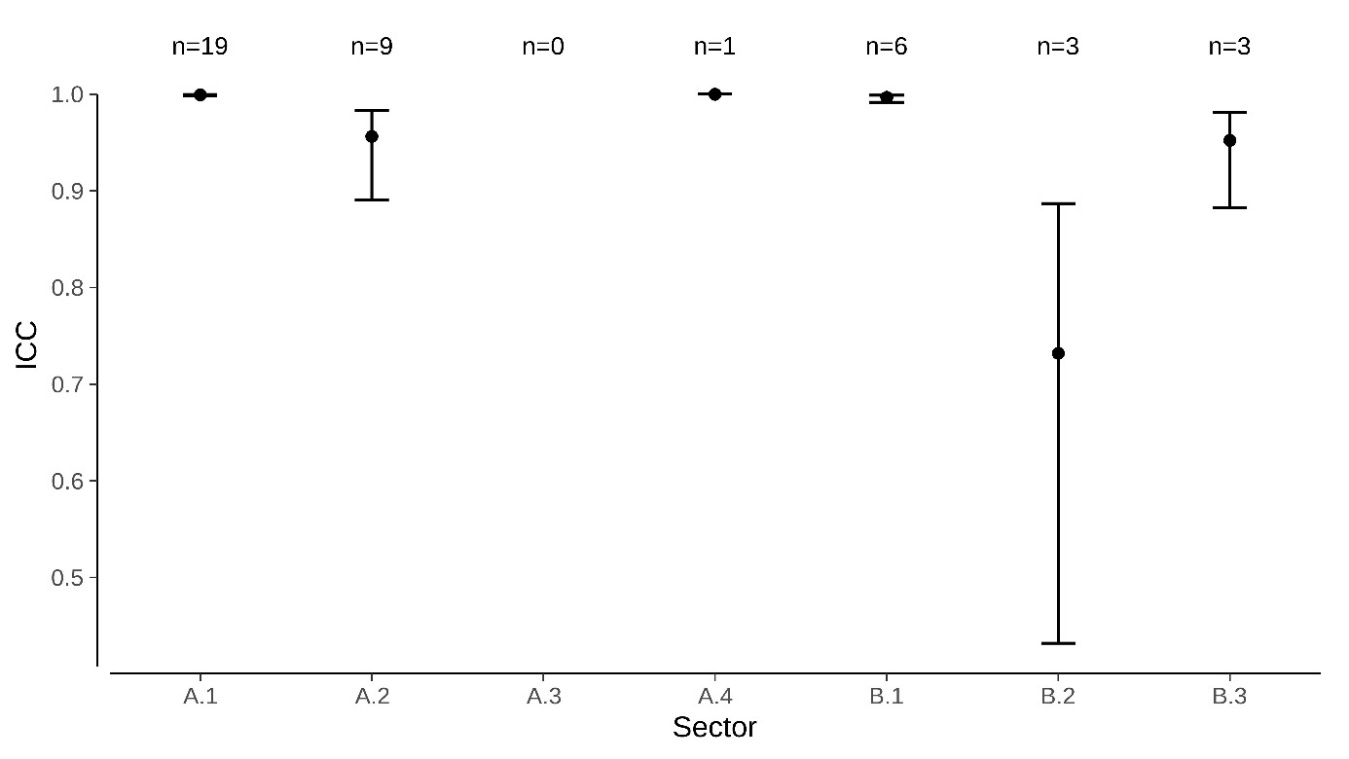


**S3 Table**. Inter Class Correlation Coefficient (ICC) with Confidence Intervals (CI) for broilers outside during instantaneous observations

| Sector | ICC (A,1) with CI | test statistics | p-value | n |
| --- | --- | --- | --- | --- |
| A.1 | 0.999; 0.998 < ICC < 1 | F(18,11.4) = 3729 | p < 0.001 | 19 |
| B.1 | 0.997; 0.992 < ICC < 0.999 | F(18,19) = 612 | p < 0.001 | 6 |
| A.2 | 0.956; 0.890 < ICC < 0.983 | F(18,18.2) = 42.6 | p < 0.001 | 9 |
| B.2 | 0.732; 0.432 < ICC < 0.887 | F(18,17.6) = 6.98 | p < 0.001 | 3 |
| A.3 |  |  |  | 0 |
| B.3 | 0.952; 0.882 < ICC < 0.981 | F(18,19) = 40.8 | p < 0.001 | 3 |
| A.4 |  |  |  | 1 |

**S4 Table.** Pearson correlation coefficient (r(80), p-value, conf. level = 0.95) between different weather indicators, i.e. wind speed, air temperature, relative humidity, solar radiation and range use (% of total broilers found outside) or maintenance behaviors (lying, standing, locomotion, foraging).

|  | Wind speed | Air temperature | Rel. humidity | Solar radiation |
| --- | --- | --- | --- | --- |
| Outside | 0.21 (0.007) | 0.52 (<0.001) | -0.51 (<0.001) | 0.01 (0.007) |
| Lying | -0.16 (0.120) | 0.52 (<0.001) | -0.49 (<0.001) | -0.05 (0.642) |
| Standing | -0.02 (0.824) | -0.20 (0.062) | 0.24 (0.024) | 0.12 (0.250) |
| Locomotion | 0.01 (0.945) | -0.10 (0.346) | 0.15 (0.173) | 0.18 (0.086) |
| Foraging | 0.17 (0.103) | -0.40 (<0.001) | 0.33 (0.001) | -0.06 (0.606) |

**S5 Table.** Average live and slaughter weights per group and trial during the trials on pasture (Weight (mean ± sem), p-value (paired t-test), n = 5). Animals were moved to pasture at the age of 30 days and weighed every two weeks individually.

| Age (days) | Treatment | Control | Statistical test | p-value |
| --- | --- | --- | --- | --- |
| 30 | 0.83 ± 0.04 | 0.83 ± 0.04 | *t(4) = 0.28* | *0.793* |
| 45 | 1.46 ± 0.08 | 1.48 ± 0.07 | *t(4) = -0.97* | *0.386* |
| 59 | 2.34 ± 0.11 | 2.30 ± 0.08 | *t(4) = 0.66* | *0.545* |
| 73 | 3.13 ± 0.07 | 2.98 ± 0.09 | *t(4) = 3.13* | *0.035* |
| Carcass weight | 2.12 ± 0.05 | 2.03 ± 0.05 | *t(4) = 3.64* | *0.022* |
